# LDGIdb: a database of gene interactions inferred from long-range strong linkage disequilibrium between pairs of SNPs

**DOI:** 10.1186/1756-0500-5-212

**Published:** 2012-05-02

**Authors:** Ming-Chih Wang, Feng-Chi Chen, Yen-Zho Chen, Yao-Ting Huang, Trees-Juen Chuang

**Affiliations:** 1 Genomics Research Center, Academia Sinica, Taipei, 11529, Taiwan; 2 Division of Biostatistics and Bioinformatics, Institute of Population Health Sciences, National Health Research Institutes, Miaoli County, 350, Taiwan; 3 Department of Life Science, National Chiao-Tung University, Hsinchu, 300, Taiwan; 4 Department of Dentistry, China Medical University, Taichung, 404, Taiwan; 5 Department of Computer Science and Information Engineering, National Chung Cheng University, Chia-yi County, 600, Taiwan

## Abstract

**Background:**

Complex human diseases may be associated with many gene interactions. Gene interactions take several different forms and it is difficult to identify all of the interactions that are potentially associated with human diseases. One approach that may fill this knowledge gap is to infer previously unknown gene interactions via identification of non-physical linkages between different mutations (or single nucleotide polymorphisms, SNPs) to avoid hitchhiking effect or lack of recombination. Strong non-physical SNP linkages are considered to be an indication of biological (gene) interactions. These interactions can be physical protein interactions, regulatory interactions, functional compensation/antagonization or many other forms of interactions. Previous studies have shown that mutations in different genes can be linked to the same disorders. Therefore, non-physical SNP linkages, coupled with knowledge of SNP-disease associations may shed more light on the role of gene interactions in human disorders. A user-friendly web resource that integrates information about non-physical SNP linkages, gene annotations, SNP information, and SNP-disease associations may thus be a good reference for biomedical research.

**Findings:**

Here we extracted the SNPs located within the promoter or exonic regions of protein-coding genes from the HapMap database to construct a database named the Linkage-Disequilibrium-based Gene Interaction database (LDGIdb). The database stores 646,203 potential human gene interactions, which are potential interactions inferred from SNP pairs that are subject to long-range strong linkage disequilibrium (LD), or non-physical linkages. To minimize the possibility of hitchhiking, SNP pairs inferred to be non-physically linked were required to be located in different chromosomes or in different LD blocks of the same chromosomes. According to the genomic locations of the involved SNPs (i.e., promoter, untranslated region (UTR) and coding region (CDS)), the SNP linkages inferred were categorized into promoter-promoter, promoter-UTR, promoter-CDS, CDS-CDS, CDS-UTR and UTR-UTR linkages. For the CDS-related linkages, the coding SNPs were further classified into nonsynonymous and synonymous variations, which represent potential gene interactions at the protein and RNA level, respectively. The LDGIdb also incorporates human disease-association databases such as Genome-Wide Association Studies (GWAS) and Online Mendelian Inheritance in Man (OMIM), so that the user can search for potential disease-associated SNP linkages. The inferred SNP linkages are also classified in the context of population stratification to provide a resource for investigating potential population-specific gene interactions.

**Conclusion:**

The LDGIdb is a user-friendly resource that integrates non-physical SNP linkages and SNP-disease associations for studies of gene interactions in human diseases. With the help of the LDGIdb, it is plausible to infer population-specific SNP linkages for more focused studies, an avenue that is potentially important for pharmacogenetics. Moreover, by referring to disease-association information such as the GWAS data, the LDGIdb may help identify previously uncharacterized disease-associated gene interactions and potentially lead to new discoveries in studies of human diseases.

**Keywords:**

Gene interaction, SNP, Linkage disequilibrium, Systems biology, Bioinformatics

## Background

Gene interactions are usually inferred from biological interactions such as protein-protein interactions (PPIs) [1-3], co-expression of genes [4,5], co-localization of proteins [6,7], co-evolution of proteins [8,9], and shared gene-phenotype associations [10]. Gene interactions that are implicated in human disorders are of particular interest [11]. Recently, it has been proposed that the associations between mutations and human disorders can be evaluated at the systems level [11-13]. This concept is based on observations that mutations in different genes can be linked to the same disorders, and that multiple mutations in the same genes can be associated with different diseases [11]. In other words, a human disorder may be the outcome of a molecular system where mutations in different genes are interconnected via a variety of gene interactions. Single nucleotide polymorphisms (SNPs) are frequently associated with human phenotypes, and SNPs in different genes that are strongly correlated with each other may be important for gene interactions. Therefore, exploring the linkages between SNPs may offer new insights into the biological interactions in the human molecular system. A database that stores information about non-physical SNP linkages and possible SNP-disease associations may be helpful for exploring the role of gene interactions in human disorders.

Here we infer potential gene interactions on the basis of long-range linkage disequilibrium (LRLD) between SNPs. We term these potential interactions “linkage disequilibrium-based gene interactions” (LDGIs), where two genes are considered to be connected if the SNPs located in these two genes are subject to strong linkage disequilibrium (LD; usually measured by *r*^*2*^ or *D′*[14]). Theoretically, LD should be observed between SNPs that are physically close to each other owing to the hitchhiking effect or lack of recombination [15]. In this study, however, we consider only the SNP pairs (designated as LRLD-SNP pairs) that are subject to strong LD (*r*^*2*^ ≥ 0.8) but are located in different LD blocks (or different chromosomes) to minimize the possibilities of accidentally linked SNPs or physical linkage, and thus increase the probability that the associations between the LRLD-linked SNPs/genes are functionally meaningful. To facilitate research based on these inferred SNP linkages (and potential gene interactions), we constructed a user-friendly database, the LDGIdb, to store the information. The LDGIdb also contains information about disease-associated SNPs/genes, such as the associations identified in genome-wide association studies (GWAS) [16] and those recorded in Online Mendelian Inheritance in Man (OMIM) database [17]. Users can thus search for LDGIs that involve disease-associated SNPs/genes, and identify potentially uncharacterized disease-associated gene interactions for further studies.

## Findings

### Construction of LDGIs

**Figure 1:**
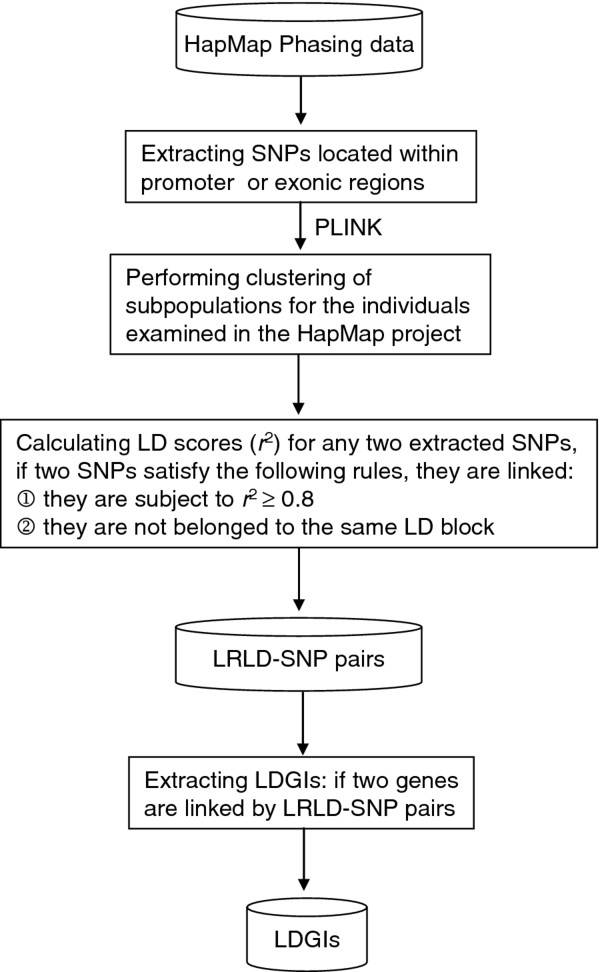
Process of identification of LRLD-SNP pairs and LDGIs.

The data analysis workflow is shown in Figure 1. We first extracted human haplotypes from the HapMap Phase II and III data [18], which were generated using the PHASE software [19]. Only the SNPs that are located within the promoter or exonic regions of protein-coding genes (with reference to the Ensembl annotations [20]) were considered. Note that the promoter regions encompass 2 kb sequences upstream of the transcriptional start sites, and exonic regions include coding regions (CDSs) and untranslated regions (UTRs). In view of population stratification, we clustered the individuals examined in the HapMap Phase II and III projects into subpopulations using the PLINK package (version 1.07) [21] (Table 1). Here we consider only the subpopulations that contain at least 20 individuals. For each subpopulation, we calculated LD scores (i.e., *r*^2^ and *D′*[14]) for all combinations of SNP pairs. Two SNPs were considered to be a long-range LD-linked SNP pair (designated as an “LRLD-SNP pair”) if they satisfied both of the following criteria: (1) to avoid the inclusion of accidentally linked SNPs, an LRLD-SNP pair had to be subject to a strong LD (*r*^2^ ≥ 0.8); (2) to minimize the probability of hitchhiking or lack of recombination, the two SNPs had to be located in different chromosomes or be separated by at least one recombination hotspot retrieved from the International HapMap Project. The latter criterion may considerably decrease the probability that the identified LRLD-SNP pairs belong to the same “LD blocks” (or “haplotype blocks”, which represent regions where recombination events occur rarely, and consequently LD is maintained) even if they are located in the same chromosomes. Accordingly, we identified 801,340 LRLD-SNP pairs, which contained 94,876 SNPs (Table 1). Genes connected by these LRLD-SNP pairs were considered human LD-based gene interactions (LDGIs). The LDGIdb is composed of a collective total of about 646,203 gene linkages, which contain 21,240 genes (Table 1). Since population stratification was also considered, the LDGIdb also provides potential population-specific gene interactions, which may be useful for investigations of population-specific traits/diseases.

**Table 1:** **Identified LRLD-SNP pairs and LDGIs (with** ***r***^***2***^**≥0.8)**

**Population**	**# Individuals**	**# LRLD-SNP pairs**	**# Affected SNPs**	**# LDGIs**	**# Affected genes**
**PLINK (*P*** **< 0.01)**					
**Phase II**					
CEU	30	66343	44756	23425	14444
**Phase III**					
CEU cluster 1	27	34940	28817	18569	11644
CEU cluster 2	40	30353	28082	15333	11308
CHD cluster 1	22	44513	29109	24675	11934
CHD cluster 2	31	30981	27563	15425	11161
JPT + CHB cluster 1	28	34672	28024	18212	11401
JPT + CHB cluster 2	22	48626	29360	29751	12014
LWK	23	42305	23398	35808	10545
MKK cluster 1	28	28924	22740	21185	10056
MKK cluster 2	24	54795	25086	47884	11203
MKK cluster 3	21	98150	27043	89007	11952
MKK cluster 4	22	63718	25598	53623	11465
YRI cluster 1	29	19116	20874	13011	9246
YRI cluster 2	28	22127	21138	16279	9390
**PLINK (*P*** **< 0.001)**					
**Phase II**					
CEU	48	61699	43945	21112	14181
JPT + CHB	20	117251	44754	61435	14792
YRI cluster 1	22	86494	38786	65698	13775
YRI cluster 2	21	98239	39389	75825	13950
**Phase III**					
ASW	25	18880	21631	11922	9432
CEU cluster 1	62	29924	27834	14997	11208
CEU cluster 2	41	30586	27967	15302	11269
CHD cluster 1	33	31456	27684	15283	11224
CHD cluster 2	21	31931	27598	15821	11215
CHI cluster 1	23	48723	30147	29355	12304
CHI cluster 2	21	56926	30705	37185	12543
JPT+CHB cluster 1	28	34509	28021	19004	11404
JPT+CHB cluster 2	30	33894	28111	17153	11424
JPT+CHB cluster 3	27	36917	28193	19958	11495
JPT+CHB cluster 4	23	44475	29077	25505	11928
JPT+CHB cluster 5	61	32011	27861	15491	11229
LWK cluster 1	21	61580	24684	54472	11194
LWK cluster 2	33	15850	19800	10436	8729
MEX	25	36330	29249	22032	11998
MKK cluster 1	41	17272	20819	10688	8997
MKK cluster 2	26	30057	22480	24739	10124
MKK cluster 3	25	52057	25081	44570	11104
MKK cluster 4	27	36459	23314	28450	10368
MKK cluster 5	24	41993	24359	33284	10779
TSI cluster 1	32	31021	28490	16191	11517
TSI cluster 2	30	32289	28501	16722	11535
YRI cluster 1	31	18261	20636	12029	9185
YRI cluster 2	37	15825	19577	9889	8602
YRI cluster 3	22	45628	23996	39632	10846
**Sum**		801340	94876	646203	21240

### Calculation of *r*^2^ and *D′* values

Let *P*_*A*_ and *P*_*B*_ be the major allele frequencies at SNP_1_ and SNP_2_, respectively. Define *P*_*a*_ and *P*_*b*_ as the minor allele frequencies at SNP_1_ and SNP_2_, respectively. Let *P*_*AB*_ be the haplotype frequency of observing both A and B alleles at these two loci. Define *D* = *P*_*AB*_ - *P*_*A*_*P*_B_*.* The LD scores, *r*^2^ and *D′*[14], between SNP_1_ and SNP_2_ can be computed by

(1) $$r^{2}=\frac{{\left(P_{\mathrm{AB}}-P_{A}P_{B}\right)}^{2}}{P_{A}(1-P_{A})P_{B}(1-P_{B})}=\frac{D^{2}}{P_{A}(1-P_{A})P_{B}(1-P_{B})}\text{,}\text{and}\;D^{\prime }=\{\begin{array}{c} \frac{D}{\min(P_{A}P_{B},P_{a}P_{b})},\text{ if }D<0;\\ \frac{D}{\min(P_{A}P_{b},P_{a}P_{B})},\text{ if }D>0. \end{array}$$

### Data retrieval

HapMap Phase II (release 22) and III (release 2) haplotype data and the corresponding recombination hotspot information were retrieved from the International HapMap Project [22]. The human protein-coding genes were downloaded from the Ensembl genome browser (release 53). The human PPI data (designated as “collected PPIs” in the LDGIdb) were collected from seven experiment-supported PPI databases: HPRD [23], DIP [24], MINT [25], IntAct [26], REACTOME [27], BioGRID [28], and MIPS [29]. The extracted PPI collection included a total of 76,955 interactions. The CRG (Centre for Genomic Regulation) human interactomes (designated as “CRG PPIs” in the LDGIdb) were downloaded from Bossi and Lehners’ study [30], which comprised 80,922 interactions. Human gene co-expression data were downloaded from the TMM database [4], which contained 203,043 high-confidence co-expression links that were observed in at least three microarray data sets. The biological interactions inferred from the above databases (i.e., collected PPIs, CRG PPIs, and co-expression links) were integrated into the LDGIdb for comparison with LDGIs. If an LDGI was not found in any of the databases, it was considered to be a potentially uncharacterized gene interaction. The GWAS [16] data were downloaded on August 23rd, 2011 [31]. For LRLD-linked genes, more detailed information was provided including protein domain descriptions (according to Interpro [32], SMART, and PFAM), KEGG pathways [33], and disease association information (OMIM, HIV interaction, and the Genetic Association Database [34]), which were all downloaded from the DAVID knowledgebase [35].

### Web interface

**Figure 2:**
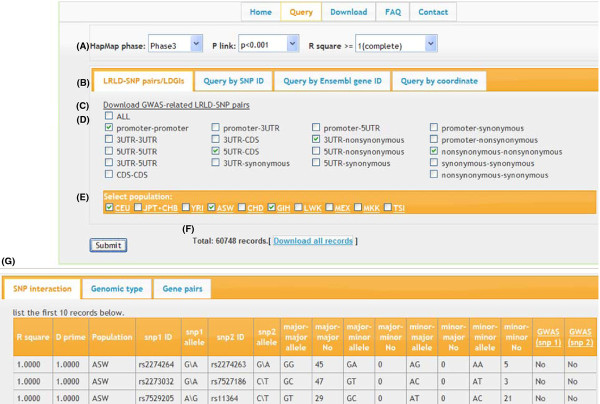
**The LDGIdb interface.(A)** The three adjustable parameters. Users can search for LRLD-SNP pairs and LDGIs by setting the three adjustable parameters: HapMap Phase (II or III), *P* value of PLINK population clustering (*P* < 0.01 or *P* < 0.001), and *r*^*2*^ value for linkage disequilibrium (≥ 0.8, ≥ 0.9, and 1). **(B)** Types of queries. Users can query by selecting the genomic types of the LRLD-linked SNP loci **(D)** and the population of interest (E), SNP accession number, gene accession number, or the coordinates of the genomic region of interest. **(C)** GWAS-related LRLD-SNP pairs. **(F)** and **(G)** are results. Users can download all records by clicking on the button **(F)**. The first 10 records are displayed **(G)**. If the linked SNP(s) is located within alternatively spliced genomic regions or overlapping genes, a LRLD-SNP pair record appears more than once with different genomic types or gene accession numbers in the downloaded file.

Users can search for LRLD-SNP pairs and LDGIs (which are linked by LRLD-SNP pairs) by setting three adjustable parameters: HapMap data source (Phase II or III), *P* value for PLINK population clustering (*P* < 0.01 or *P* < 0.001), and *r*^*2*^ value for linkage disequilibrium (≥0.8, ≥0.9, or 1) (Figure 2A). Note that we only considered population clusters containing at least 20 individuals (Table 1). Also note that LDLR-SNP pairs with *r*^*2*^ = 1 are subject to a “complete” LD. The LDGIdb supports four types of queries. Users can search for LRLD-SNP pairs/LDGIs by specifying the types of genomic location of LRLD-linked SNPs, SNP ID, gene accession number(s), or genomic coordinates (Figure 2B). GWAS-related LRLD-SNP pairs are also provided (Figure 2C). As shown in Figure 2D, the LRLD-SNP pairs/LDGIs are categorized, according to the types of genomic location of the linked SNPs, into promoter-promoter, promoter-UTR, promoter-CDS, CDS-CDS, CDS-UTR and UTR-UTR interactions. The CDS-related LDGIs are further categorized according to whether the LD-linked SNPs are nonsynonymous or synonymous (Figure 2D). Therefore, the user can choose LRLD-SNP pairs that occur in different genomic regions and that (in the case of coding SNPs) represent changes at the RNA or protein levels (the user can choose more than one type of interaction). The user can further select one or more population of interest to retrieve population-specific LDGIs. The results are downloadable (Figure 2E). For simplicity, the web interface displays only the first 10 records of each query (Figure 2F). The user can find detailed information of allele combinations of LRLD-linked SNPs and genomic regions where the linked SNPs are located in the results (Figure 2G). For the identified LDGIs, the interface also provides human PPI data collected from eight experiment-supported databases (i.e., collected PPIs and CRG PPIs) and high-confidence co-expression interactions for comparison. More detailed information of LDGI genes is also provided, including protein domain annotations, biological pathways, and disease associations.

### Discussion and future development

Here we propose a new resource for studies of potential human gene interactions (i.e., LDGIs) based on haplotype data. In LDGIs, the linked genes are located in different chromosomes or LD blocks but are connected by one or more exonic/promoter SNP pairs that are subject to strong linkage disequilibrium (*r*^*2*^ ≥ 0.8, ≥ 0.9, or 1). We suggest that this LRLD approach and the LDGIdb can be potentially applied to the following areas. First, LDGIs may represent potential uncharacterized gene interactions, in which the functional associations between the LDGI genes may not be explicitly indicated in other biological networks. Second, although we constructed the LDGIdb using SNP data in this study, the LRLD approach can actually be expanded to include other types of genomic variants such as copy number variation and insertion/deletion. Third, given enough haplotype information, population-specific LDGIs/LRLD-SNP pairs may be identified for more focused studies, particularly in the field of pharmacogenetics. Fourth, the correlation between the LDGIs/LRLD-SNP pairs and disease-associated SNPs such as those identified in GWAS studies can be explored. For example, SNP rs393152, which is associated with Parkinson’s disease [36], forms an LRLD-SNP pair with rs12185268. Interestingly, rs12185268 was demonstrated to be connected to the same disease [37] two years after the publication (i.e., Ref #36) of the association of rs393152 with the disease. Another example is the LRLD-SNP pair: rs9858542–rs3197999. The two SNPs in this pair were shown to be related, respectively, to the Crohn’s disease [38-41] and the ulcerative colitis [42,43]. These examples show that two SNPs that are associated with the same (or related) human diseases/traits can be identified by our approach. Moreover, there are also cases in which GWAS SNPs and their LDGI partners are associated with the same (or related) human diseases. For example, the GWAS SNP rs5215 in *KCNJ11* is known to be associated with Type II diabetes [44,45]. This SNP forms an LRLD-SNP pair with rs757110, which is located within the CDS of *ABCC8*. Mutations and deficiencies in the protein encoded by *ABCC8* have been suggested to be associated with hyperinsulinemic hypoglycemia of infancy and non-insulin-dependent diabetes mellitus type II [46,47]. The above examples suggest that the LRLD-SNP linkages may reflect biological interactions in the human molecular system and have the potential to detect previously uncharacterized gene interactions. As disease-association data accumulate, the LDGIdb may become an increasingly powerful tool by which to identify potentially uncharacterized disease-associated gene interactions, contributing to network-based studies of human diseases. Notably, however, since the majority of HapMap SNPs are relatively common variants, the linkages of rare alleles may not be represented in LDGIdb.

This study actually examined whether observed non-physical SNP linkages occur simply by chance or whether they are biologically meaningful. The above examples suggest that the inferred LDGIs may be functionally relevant. One interesting question is what are the molecular mechanisms underlying the inferred gene interactions. For the CDS-CDS LDGIs that involve only nonsynonymous changes, the functional association is speculated to result from direct or indirect protein-level interactions. Of course, the LDGIs may also represent adventitious linkages or false positives that result from unknown population substructures. Meanwhile, the biological meanings of the LDGIs that involve UTR SNPs (i.e., CDS-UTR and UTR-UTR linkages) or synonymous SNPs (i.e., nonsynonymous-synonymous and synonymous-synonymous linkages) may be more subtle. These potential interactions may be associated with translational regulation. Specifically, 5′UTRs may contain multiple sequence features that are involved in translational regulation, including upstream open reading frames, secondary structures, internal ribosome entry sites, and iron regulatory protein binding sites [48]. The disruption of these functional elements may cause changes in the efficiency of protein translation. On the other hand, 3′UTRs are known to be the major binding target of microRNAs, which can also suppress protein expression [49]. In addition, 3′UTRs may harbor protein-interacting secondary structures or the signals of nonsense-mediated decay or polyadenylation [48], both of which can affect the efficiency of protein translation. Meanwhile, synonymous coding SNPs are known to affect mRNA stability and splicing, leading to changes in the corresponding protein products [50]. Since both the UTR and synonymous SNPs may affect protein abundance, dosage imbalance and unidentified, indirect protein interactions may be possible explanations for the observed linkages.

## Competing interests

The authors declare that they have no competing interests.

## Authors’ contributions

TJC conceived and designed the study. FCC, YTH and TJC conducted the analyses. MCW and YZC built the web server. FCC and TJC wrote the manuscript. All authors read and approved the final manuscript.

## Availability and requirements

Project name: LDGIdb project

Availability: LDGIdb is freely accessible at http://LDGIdb.genomics.sinica.edu.tw. Operating systems: Platform independent

Programming language: Javascript, CSS, PHP

Other requirements: None
